# CD18 controls the development and activation of monocyte-to-macrophage axis during chronic schistosomiasis

**DOI:** 10.3389/fimmu.2022.929552

**Published:** 2022-10-03

**Authors:** Camila O. S. Souza, Jefferson Elias-Oliveira, Marcella R. Pastore, Caroline Fontanari, Vanessa F. Rodrigues, Vanderlei Rodriguez, Luiz G. Gardinassi, Lúcia H. Faccioli

**Affiliations:** ^1^ Departamento de Análises Clínicas, Toxicológicas e Bromatológicas, Faculdade de Ciências Farmacêuticas de Ribeirão Preto, Universidade de São Paulo, Ribeirão Preto, Brazil; ^2^ Programa de Pós-Graduação em Imunologia Básica e Aplicada, Faculdade de Medicina de Ribeirão Preto, Universidade de São Paulo, Ribeirão Preto, Brazil; ^3^ Programa de Pós-Graduação em Biociências e Biotecnologia Aplicadas à Farmácia, Faculdade de Ciências Farmacêuticas de Ribeirão Preto, Ribeirão Preto, Brazil; ^4^ Departamento de Bioquímica e Imunologia, Faculdade de Medicina de Ribeirão Preto, Universidade de São Paulo, Ribeirão Preto, Brazil; ^5^ Departamento de Biociências e Tecnologia, Instituto de Patologia Tropical e Saúde Pública, Universidade Federal de Goiás, Goiânia, Brazil

**Keywords:** β2 integrin, monocytes, proliferation, alternatively activated macrophages, schistosomiasis

## Abstract

Schistosomiasis is a neglected tropical disease caused by worms of the genus *Schistosoma* spp. The progression of disease results in intense tissue fibrosis and high mortality rate. After egg deposition by adult worms, the inflammatory response is characterized by the robust activation of type 2 immunity. Monocytes and macrophages play critical roles during schistosomiasis. Inflammatory Ly6C^high^ monocytes are recruited from the blood to the inflammatory foci and differentiate into alternatively activated macrophages (AAMs), which promote tissue repair. The common chain of β_2_-integrins (CD18) regulates monocytopoiesis and mediates resistance to experimental schistosomiasis. There is still limited knowledge about mechanisms controlled by CD18 that impact monocyte development and effector cells such as macrophages during schistosomiasis. Here, we show that *CD18^low^* mice chronically infected with *S. mansoni* display monocyte progenitors with reduced proliferative capacity, resulting in the accumulation of the progenitor cell denominated proliferating-monocyte (pMo). Consequently, inflammatory Ly6C^high^ and patrolling Ly6C^low^ monocytes are reduced in the bone marrow and blood. Mechanistically, low CD18 expression decreases *Irf8* gene expression in pMo progenitor cells, whose encoded transcription factor regulates CSFR1 (CD115) expression on the cell surface. Furthermore, low CD18 expression affects the accumulation of inflammatory Ly6C^high^ CD11b^+^ monocytes in the liver while the adoptive transference of these cells to infected-*CD18^low^* mice reduced the inflammatory infiltrate and fibrosis in the liver. Importantly, expression of *Il4*, *Chil3l3* and *Arg1* was downregulated, CD206^+^PD-L2^+^ AAMs were reduced and there were lower levels of IL-10 in the liver of *CD18^low^* mice chronically infected with *S. mansoni*. Overall, these findings suggest that CD18 controls the IRF8-CD115 axis on pMo progenitor cells, affecting their proliferation and maturation of monocytes. At the same time, CD18 is crucial for the appropriate polarization and function of AAMs and tissue repair during chronic schistosomiasis.

## Introduction

The common chain of β_2_ integrins (CD18) is constitutively expressed by diverse leukocytes and is involved in host-pathogen interactions, adhesion and trans-endothelial migration of leukocytes, phagocytosis and killing of pathogens, and monocytopoiesis during schistosomiasis (1–3). Mice that express low levels of CD18 (*CD18^low^*) exhibit reduced accumulation of inflammatory Ly6C^high^ and patrolling Ly6C^low^ monocytes in the bone marrow (BM) and blood during infection with *S. mansoni*, which correlates with increased egg counts on feces and higher mortality (3). The accumulation of *S. mansoni-*eggs into tissue induces high levels of IL-4, IL-13, IL-5, and IL-10 that trigger Th2 granuloma formation, a hallmark of chronic schistosomiasis (4). Granulomas are structures composed by non-immune and immune cells which are recruited to limit the tissue damage caused by eggs (5). In general, the cellular infiltrate in liver from *S. mansoni-*infected mice is characterized by increased numbers of monocytes, neutrophils, eosinophils and macrophages around the parasites eggs (6). Low CD18 expression does not affect the accumulation of neutrophils or monocyte-derived macrophages (MDM) and monocyte-derived dendritic cells (MDC) in the liver, however, it reduces specific monocyte subsets during infection with *S. mansoni* (3). Therefore, we hypothesized that CD18 is required by progenitor cells as well as by mature monocytes to resist immunopathology caused by *S. mansoni* infection.

Leukocyte adhesion deficiency type 1 (LAD1) is caused by mutations on *ITGB2* (CD18) gene in humans, which are affected by recurrent bacterial and fungal infections (7). Patients with chronic granulomatous disease (CGD) express reduced levels of CD18, which are associated with impaired innate immune cell recruitment and uncontrolled inflammatory responses (8). Previous studies show that CD18 knockout (*CD18^-/-^*) or CD18 hypomorphic (CD18^HYP^) mice exhibit increased numbers of Sca-1^+^ c-Kit^+^ cells (LSK pools) and increased neutrophils in the bone marrow (9, 10), suggesting that CD18 regulates the proliferation of hematopoietic stem and progenitor cells (HSPCs) that form hematopoietic niches (11, 12). During *S. mansoni* infection, lower CD18 expression does not affect neutrophil hematopoiesis in the bone marrow (3). CD18 controls the proliferation of granulocytes/macrophages progenitors (GMP) (9), suggesting a role for proliferative precursors during monocytopoiesis (13, 14). In adult life, the development of monocytes is initiated from the common bone marrow-derived precursor called the ‘monocyte-macrophages DC progenitor’ (MDP), followed by sequential steps of ‘common monocyte progenitor’ (cMoP) that give rise to the proliferating-monocyte (pMo) progenitor, which matures into monocytes (14).

After *S. mansoni* infection, most macrophages at inflammatory sites are derived from blood monocytes (6, 15–17). Both tissue resident macrophages and MDMs become alternatively activated macrophages (AAMs) *via* IL-4Rα signaling by IL-4 and IL-13 (15, 18). In general, the alternative phenotype is characterized by expression of high levels of arginase-1 (*Arg1)*, chitinase-like 3 (*Chi3l3)*, resistin-like alpha (*Relma)* and mannose receptor C-type 1 *(Mrc1) (* 19). However, only monocyte-derived AAMs upregulates the expression of retinaldehyde dehydrogenase 2 (*Raldh2)* and programmed cell death ligand 2 (*Pdcd1lg2*) (20–22). Furthermore, AAMs produce IL-10 and TGF-β (23), cytokines that promote healing and tissue repair during schistosomiasis (24, 25). On the other hand, the integrin formed by pairing of CD11b – CD18 (αM/β2), MAC-1, forms a complex with IL-13Rα1 on the surface of macrophages (26) and dampens the alternative activation during the chronic inflammation (27).

To investigate the molecular role of CD18 on monocyte progenitor cells and alternative activation of macrophages during schistosomiasis, we used a mice model that expresses low levels of CD18 (*CD18^low^)*, thus resembling humans with moderate ITGB2 deficiency (28). We found that CD18 affects the expression of the proliferation marker, Ki-67 and regulates the proliferation of monocyte progenitors during infection by *S. mansoni*. Gene expression of *Irf8* was reduced in pMo cells of infected-*CD18^low^* mice, and consequently affected CD115 expression in the cell surface, resulting in failure of monocyte maturation in the BM and blood. The frequency of inflammatory Ly6C^high^ CD11b^+^ monocytes was reduced in the liver of infected *CD18^low^* mice and the adoptive transference of these cells ameliorated the inflammatory infiltrate and tissue fibrosis, independently of the parasite burden. Of importance, CD18 regulates the expression of genes involved in alternative activation, such as *Il4*, *Chil3l3* and *Arg1* and impact CD206^+^ PD-L2^+^ AAMs, which was associated with lower levels of IL-10 in the liver. These data fill important gaps about the molecular mechanisms driven by CD18 during monocytopoiesis and alternative activation of macrophages in response to infection by *S. mansoni*.

## Materials and methods

### Animals

Male 8-week-old (22-26g) C57BL/6 (wild-type, WT) and homozygous *CD18^low^* mice on the C57BL/6 background were obtained from the animal facilities of the Faculdade de Ciências Farmacêuticas de Ribeirão Preto, Universidade de São Paulo (FCFRP – USP). *CD18^low^* (B6.129S-Itgb2^tm1bay^) mice were purchased at The Jackson Laboratory. The *CX_3_CR1^gfp/gfp^* mice on the C57BL/6 background was donate by Dr. João Santana Silva from Faculdade de Medicina de Ribeirão Preto, Universidade de São Paulo (FMRP – USP). To obtain *CX_3_CR1^gfp/+^* mice, *CX_3_CR1^gfp/gfp^* animals were backcrossed for ten generations with C57BL/6 mice. All experiments using animals were approved by the Comissão de Ética no Uso de Animais da Faculdade de Ciências Farmacêuticas de Ribeirão Preto (Protocol Number 14.1.607.53.9 and 19.1.46.60.4) and carried out in accordance to the ethical principles for animal research adopted by the Sociedade Brasileira de Ciência em Animais de Laboratório.

### Parasite maintenance and experimental infection

*Schistosoma mansoni* LE strain was maintained by routine passage through *Biomphalaria glabrata* snails and BALB/c mice (20-25g) from the animal facilities of the Faculdade de Medicina de Ribeirão Preto - Universidade de São Paulo (FMRP – USP). Recovery of cercariae was described previously (3). For infection, mice were subcutaneously inoculated with 80 cercariae/animal with a sterile syringe and 22 G x 1” needle (BD Biosciences, Franklin Lakes, New Jersey, USA). At 7 weeks post infection (wpi) the animals were euthanized for posterior analyses.

### Quantification of worm’s burden and fecal eggs

Parasite burdens were recovered by portal perfusion with 20 mL/animal of perfusion buffer [58 mM Na_3_C_6_H_5_O_7_ and 145 mM NaCl (Sigma Aldrich, St. Louis, Missouri, USA)] at 37°C. The worms were washed and counted using a dissecting microscope. The fecal eggs were recovered in stool sample/animal by Kato-Katz technique, as previously described (29).

### Histopathological analysis

Animals from each experimental group were euthanized at 7 wpi. The liver was excised, fixed with 10% formalin for 24h, and embedded in paraffin. The tissue sections (5μm) were stained with H&E and Picrosirius red coloration for evaluate inflammatory infiltrated and fibrosis, respectively. Images were captured with a digital video camera (Leica^®^ Microsystems, Heebrugg, Switzerland) adapted to DMR microscope (Leica^®^, Microsystems GmbH, Wetzlar, Germany). The images were processed using the Leica QWin software (Leica Microsystems Image Solution^®^, Cambridge, UK). The inflammatory infiltrated and fibrosis were quantified with Image J (V1.51) software.

### Anti-CD18 treatment *in vivo*

Naïve C57BL/6 mice were treated with anti-CD18 (clone: GAME-46) *via* intravenous route (2mg/kg of body weight) over 3 days. Controls were treated with saline buffer. Schematic representation of the treatment with anti-CD18 is shown in **Figure 3E**.

### Flow cytometry of bone marrow, blood, and liver cells

The BM was flushed out from two femurs and tibias using RPMI with help of 26 G x ½” needle. Peripheral blood was drawn from the retro-orbital plexus. The red blood cells in BM flush and blood were lysed, and remaining cells were washed in PBS containing 5% FBS, centrifuged and resuspended in RPMI 1640 containing 5% FBS. Cell suspensions were used in further analysis. Liver cells suspension were obtained after tissue digestion at 37°C for 45 min in 4 mL/liver of digestion buffer [HBSS, 0.05% collagenase II (Sigma Aldrich, St. Louis, Missouri, USA) and 1 mg/mL DNase (Sigma Aldrich, St. Louis, Missouri, USA)]. The enzymatic digestion was stopped by adding 100 μL of FBS and the tissue fragments passed through a cell strainer 100 μM pore size (Corning Inc. New York, USA). The resulting suspension was centrifuged at 1,300 rpm, 10 min, 4°C. The cellular pellet was resuspended in 40% of isotonic Percoll and centrifuged at room temperature for 30 min at 1.500 g. Next, red blood cells were lysed, and remaining cells were washed in PBS, centrifuged, and resuspended in RPMI 1640 containing 5% FBS. Suspensions of 2 x 10^6^ cells were used in further analysis. For viability, cells were counted in trypan blue or used the Fixable Viability Stain (FVS) (1:100) (BD Horizon™). The following antibodies were used: CD3 (1:100; clone: 17A2); CD19 (1:100; clone: 1D3); Ly6G (1:100; clone: 1A8); NK1.1 (1:100; clone: PK136); Ter-119/Erythroid (1:100; clone: TER-119) CD45R/B220 (1:100; clone: RA3-6B2); CD117/c-Kit (1:100; clone: 2B8); CD135 (1:100; clone: A2F10); CD115 (1:200; clone: CSF-1R); Ly6C (1:100; clone: AL-21 or HK1.4); CD11a (1:100; clone: 2D7); CD11b (1:100; clone: M1/70); CD11c (1:100; clone: HL3); Ki-67 (1:200; clone B56) CD45 (1:100; clone: 30-F11), CX_3_CR1 (1:200; clone: SA011F11), F4/80 (1:100; clone: BM8), CD206 (1:100; clone: C068C2) and CD273 / PD-L2 (1:100; clone: TY25). All antibodies used in flow cytometry were purchased from Biolegend (San Diego, CA, EUA) or BD Biosciences (Franklin Lakes, New Jersey, USA). Data acquisition was performed using a BD LSRFortessa™ flow cytometer and FACSDiva software (BD Biosciences, Franklin Lakes, New Jersey, USA). 100,000 events were acquired for samples from bone marrow, blood and liver. Data were plotted and analyzed using FlowJo software v. 10.8.0 (Tree Star, Inc, Ashland, OR, USA)

### Cell sorting from BM

Bone marrows (two femurs and tibias) from three chronically infected WT or *CD18^low^* mice were flushed out using RPMI with 26 G x ½” needle. Pooled cell suspensions were stained with Fixable Viability Stain (FVS) (1:1000) (BD Horizon™); following the antibodies: CD3 (1:100; clone: 17A2), CD19 (1:100; clone: 1D3), NK1.1 (1:100; clone: PK136) Ly6G (1:100; clone A18), CD117 / c-Kit (1:200; clone: 2B8), CD115 (1:200; clone: CSF-1R), Ly6C (1:200; clone: AL-21) and CD11b (1:200; clone M1/70) washed and after isolated using a BD FACSMelody™ Cell Sorter. Two populations were separated, proliferating-monocyte progenitor (pMo) (FVS^-^ Lin^-^ CD117^+^ CD115^+^ Ly6C^+^ CD11b^-^) and inflammatory Ly6C^high^ monocytes (FVS^-^ Lin^-^ CD117^+^ CD115^+^ Ly6C^high^ CD11b^+^). Suspensions of 1 x 10^5^ proliferating-monocyte progenitor (pMo) and 1 x 10^6^ inflammatory Ly6C^high^ monocytes were used in further analysis.

### Cell sorting of inflammatory Ly6C^high^CD11b^+^ and patrolling Ly6C^low^CD11b^+^monocytes from the BM of CX_3_CR1^gpf/+^ animals and adoptive transference

Bone marrows (two femurs and tibias) of three non-infected *CX_3_CR1^gfp/+^* mice were flushed out using RPMI and 26 G x ½” needle. Pooled cell suspensions were stained with antibodies: Ly6G (1:200; clone 1A8), Ly6C (1:200; clone AL-21) and CD11b (1:200; clone M1/70) washed and isolated using a BD FACSMelody™ Cell Sorter. Suspensions of 2 x 10^5^ inflammatory Ly6C^high^ (Ly6G^-^ Ly6C^high^ CD11b^+^ CX_3_CR1^low^) and 1 x 10^5^ patrolling Ly6C^low^ (Ly6G^-^ Ly6C^low^ CD11b^+^ CX_3_CR1^low^) monocytes were transferred by intravenous route to infected-*CD18^low^* mice in different days shown in the experimental design shown in **Figure 4A**.

### Quantitative real-time polymerase chain reaction

For BM lysates and isolated pMo and inflammatory Ly6C^high^ monocytes, the total RNA was extracted using the RNA*later* ^®^ (Sigma-Aldrich) and PureLink™ RNA Mini Kit (Invitrogen™) according to the manufacturer instructions. For liver homogenates, the total RNA was extracted using the TRIzol^®^ and the SV Total RNA Isolation System Kit (Promega^®^) according to the manufacturer instruction. Complementary DNA was synthesized with the High Capacity cDNA Reverse Transcription Kit (Applied Biosystems). SYBR Green Mix–based real-time quantitative PCR assays were performed using the StepOnePlus Real-Time PCR System (Applied Biosystems). The mean cycle threshold (Ct) values of triplicate measurements were used to calculate the expression of the target genes, which were normalized to the housekeeping gene *Gapdh* to liver and BM cells, and analyzed with the 2^-ΔΔCt^ method. All primers **(** **Supplementary Table 1** **)** were designed using the Primer Express software package v2.0 (Applied Biosystems), based on the nucleotide reference sequences available at GenBank database.

### Statistical analyses

The data were normally distributed. Significant differences between experimental groups were evaluated using one-way ANOVA and Tukey’s multiple comparison test or Student’s t-test. Error bars represent mean ± SD. Analyses were performed using GraphPad Prism software v8.2.1 (GraphPad Software Inc., San Diego, CA, USA). Statistical significance was set at p <0.05.

## Results

### CD18 affects Ki-67 expression and maturation of monocyte progenitors during schistosomiasis

**Figure 1:**
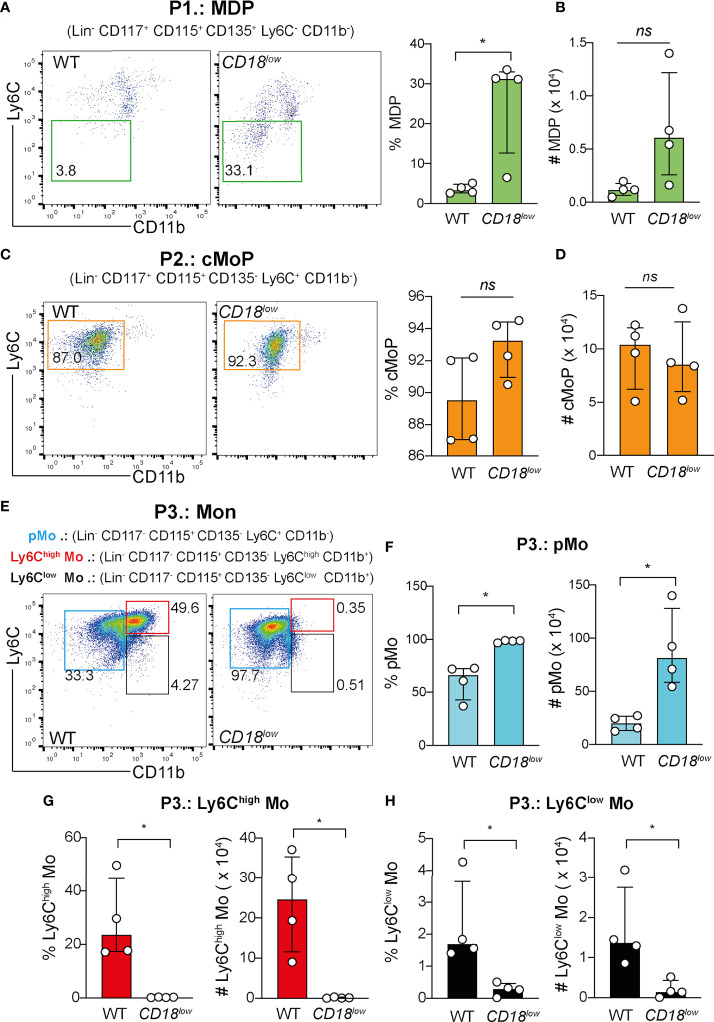
CD18 is required for monocyte progenitors in BM during chronic schistosomiasis. BM of *S. mansoni*-infected C57BL/6 and *CD18^low^* mice were analyzed by flow cytometry. **(A)** Representative dot plots and graph displaying the percentage of MDP progenitor cells (Lin^-^ CD117^-^ CD115^+^ CD135^+^ Ly6C^-^ CD11b^-^). **(B)** Scatter plot with bar show the absolute numbers of MPD progenitor cells. **(C)** Representative dot plots and graph displaying the percentage of cMoP progenitor cells (Lin^-^ CD117^+^ CD115^+^ CD135^+^ Ly6C^+^ CD11b^-^). **(D)** Scatter plot with bar show the absolute number of cMoP progenitor cells. **(E)** Representative dot plots displaying the pMo progenitor cells (Lin^-^ CD117^-^ CD115^+^ CD135^-^ Ly6C^+^ CD11b^-^), inflammatory Ly6C^high^ monocytes (Lin^-^ CD117^-^ CD115^+^ CD135^-^ Ly6C^high^ CD11b^+^) and patrolling Ly6C^low^ monocytes (Lin^-^ CD117^-^ CD115^+^ CD135^-^ Ly6C^low^ CD11b^+^). **(F)** Scatter plot with bar show the percentage and absolute numbers of pMo progenitor cells. **(G)** Scatter plot with bar show the percentage and absolute numbers of inflammatory Ly6C^high^ monocytes. **(H)** Scatter plot with bar show the percentage and absolute numbers of patrolling Ly6C^low^ monocytes. Median with interquartile range are shown for one representative experiment (n= 4 WT and *CD18^low^* infected mice at 7 weeks) out of three independent experiments. Data were analyzed with Mann-Whitney test (*p < 0.05, ns p > 0.05, compared to WT in each time-point). The symbol (#) indicates the absolute numbers of each progenitor population.

To assess the biological function of CD18 in regulating the bone marrow-derived precursors of monocytes during chronic schistosomiasis, WT and *CD18^low^* mice were infected subcutaneously with 80 cercariae and after 7 wpi, the monocytopoiesis was characterized in the BM with flow cytometry (**Figure S1A** **)**. Compared to controls, *CD18^low^* mice exhibited increased percentage of MPD **(** **Figure 1A** **)**, but absolute numbers of cells were similar **(** **Figure 1B** **)**. There were no significant differences in cMoP progenitors between experimental groups **(** **Figures 1C, D** **)**. The final steps of monocyte maturation can be characterized by different levels of Ly6C and CD11b expression: Ly6C^+^ CD11b^-^ (pMo), Ly6C^high^ CD11b^+^ (inflammatory monocytes) and Ly6C^low^CD11b^+^ (patrolling monocytes) **(** **Figure 1E** **)**. Strikingly, we observed that both percentage and absolute numbers of pMo increased in the BM of infected *CD18^low^* mice **(** **Figures 1E, F** **)**. In accordance, the percentage and absolute number of inflammatory Ly6C^high^ **(** **Figures 1E, G** **)** and patrolling Ly6C^low^ **(** **Figures 1E, H** **)** monocytes were significantly diminished in the BM of *CD18^low^* animals. We also analyzed the frequency of blood monocytes using a flow cytometric gating hierarchy including the CX_3_CR1 marker **(** **Figure S1B** **)**. Corroborating previous findings (3), the percentage and absolute numbers of inflammatory Ly6C^high^ and patrolling Ly6C^low^ monocytes were reduced in *CD18^low^* mice **(** **Figure S1B** **)**.

**Figure 2:**
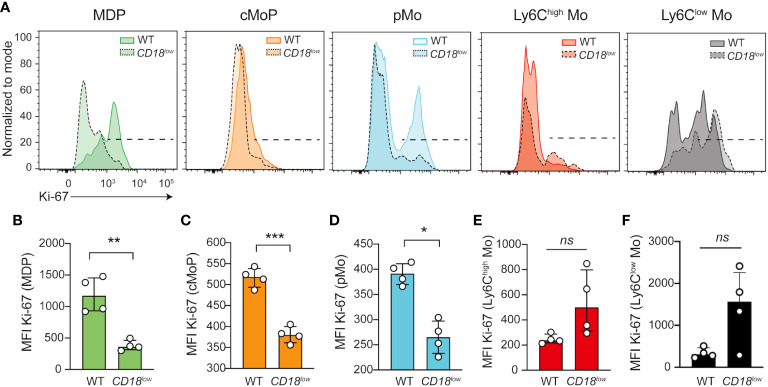
CD18 controls the expression of Ki-67 in monocyte progenitors in BM during chronic schistosomiasis. BM of *S. mansoni*-infected C57BL/6 and *CD18^low^* mice were analyzed by flow cytometry. **(A)** Representative histogram graph showed the median florescent intensity (MFI) of Ki-67 in MPD (Lin^-^ CD117^-^ CD115^+^ CD135^+^ Ly6C^-^ CD11b^-^), cMoP (Lin^-^ CD117^+^ CD115^+^ CD135^+^ Ly6C^+^ CD11b^-^) and pMo (Lin^-^ CD117^-^ CD115^+^ CD135^-^ Ly6C^+^ CD11b^-^) progenitor cells and in distinct monocytes subsets, Ly6C^high^ (Lin^-^ CD117^-^ CD115^+^ CD135^-^ Ly6C^high^ CD11b^+^) and Ly6C^low^ (Lin^-^ CD117^-^ CD115^+^ CD135^-^ Ly6C^low^ CD11b^+^). **(B)** Scatter plot with bar show MFI of Ki-67 in MPD progenitor cells. **(C)** Scatter plot with bar show Ki-67 MFI in cMoP progenitor cells. **(D)** Scatter plot with bar show MFI of Ki-67 in pMo progenitor cells. **(E)** Scatter plot with bar show MFI of Ki-67 in inflammatory Ly6C^high^ monocytes. **(F)** Scatter plot with bar show MFI of Ki-67 in patrolling Ly6C^low^ monocytes. Median with interquartile range are shown for one representative experiment (n= 4 WT and *CD18^low^* infected mice at 7 weeks) out of three independent experiments. Data were analyzed with Mann-Whitney test (* p < 0.05, ** p < 0.01, *** p < 0.001, ns p > 0.05, compared to WT in each time-point).

Furthermore, CD18 deficiency has been associated with the proliferation of myeloid cells (9, 12), suggesting that CD18 affects the proliferation of monocyte progenitors and development during chronic schistosomiasis. To test this hypothesis, we quantified the expression of the proliferation marker, Ki-67, in progenitors and mature monocytes. Compared with WT mice, Ki-67 expression was significantly reduced in MPD **(** **Figures 2A, B** **)**, cMoP **(** **Figures 2A, C** **)** and pMo **(** **Figures 2A, D** **)** progenitors in the BM of *CD18^low^* mice at 7 wpi. There were no significant differences in Ki-67 expression in inflammatory Ly6C^high^ **(** **Figures 2A, E** **)** and patrolling Ly6C^low^ **(** **Figures 2A, F** **)** monocytes. Collectively, these data suggest that low CD18 expression impacts the proliferation of monocyte precursors, a process that is required for the differentiation into mature monocytes (14). Therefore, a potential development arrest may drive the accumulation of these cells, reducing mature monocytes in the BM and blood during chronic *S. mansoni* infection.

### CD18 regulates IRF8-dependent CD115 expression in pMo progenitors during chronic schistosomiasis

**Figure 3:**
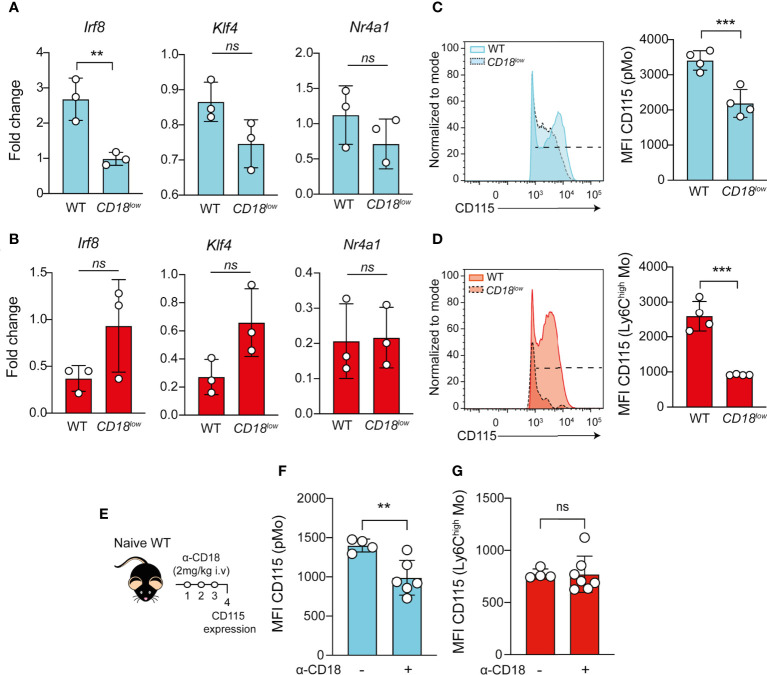
Low CD18 expression impacts the expression of IRF8 and CD115 in proliferating-monocyte isolated from BM of *S. mansoni-*infected mice. Proliferating-monocytes (pMo) progenitor cells and inflammatory Ly6C^high^ monocytes were isolated by FACS-sorting from the BM of uninfected and *S. mansoni-*infected C57BL/6 and *CD18^low^* mice. **(A)** Scatter plot with bar show the *Irf8*, *Klf4* and *Nr4a1* expression in the proliferating-monocytes progenitor cells at 7 wpi. **(B)** Scatter plot with bar show the *Irf8*, *Klf4* and *Nr4a1* expression in the inflammatory Ly6C^high^ monocytes at 7 wpi. Data represent one independent experiment using a pool of three animals (N=9 WT and *CD18^low^* infected mice at 7 weeks. Results are expressed as mean ± SD. Statistically significant differences were evaluated with unpaired t test ( ** p < 0.01 compared to WT in each time-point). BM of *S. mansoni*-infected C57BL/6 and *CD18^low^* mice were analyzed by flow cytometry. **(C)** Representative histogram and scatter plot with bar show the CD115 MFI on pMo (Lin^-^ CD117^-^ CD115^+^ CD135^-^ Ly6C^+^ CD11b^-^) progenitor cells **(D)** Representative histogram and scatter plot with bar show the CD115 MFI on inflammatory Ly6C^high^ (Lin^-^ CD117^-^ CD115^+^ CD135^-^ Ly6C^high^ CD11b^+^) monocytes. Data represent one representative experiment (n= 4 WT and *CD18^low^* infected mice at 7 weeks) out of three independent experiments. Statistically significant differences were evaluated with unpaired t test (*** p < 0.001, ns p > 0.05, compared to WT in each time-point). **(E)** Schematic representation of the treatment with anti-CD18 (intravenous 2mg/Kg) in naïve WT mice. **(F)** Scatter plot with bar show the CD115 MFI on pMo progenitor cells. **(G)** Scatter plot with bar show the CD115 MFI on inflammatory Ly6C^high^ monocytes. Data represent one independent experiment (n= 4 non treated WT mice and n=7 WT anti-CD18 treated mice per group). Statistically significant differences were evaluated with unpaired t test (** p < 0.01, ns p > 0.05, compared to nontreated WT mice).

The transcription factors IRF8 (30, 31) and KLF4 (32, 33) control the development of Ly6C^high^ monocytes, while NR4A1 (34) is necessary for the maturation of Ly6C^low^ monocytes. Because we observed the accumulation of pMo progenitors and reduced Ki-67 expression in the cells from *CD18^low^* mice, we next investigated if CD18 affects the gene expression of transcription factors controlling the maturation of monocytes. Using FACS-sorting, we isolated the pMo (Ly6C^+^CD11b^-^) and inflammatory Ly6C^high^ monocytes from the BM of naïve and chronically infected WT and *CD18^low^* mice. Compared to WT mice, *Irf8* expression was significantly reduced in the pMo cells from *CD18^low^* animals **(** **Figure 3A** **)**. There were no significant differences in *Klf4* and *Nr4a1* expression in pMo cells between the mouse strains **(** **Figure 3B** **)**. Inflammatory Ly6C^high^ monocytes from both experimental groups displayed similar expression of *Irf8, Klf4* and *Nr4a1*. IRF8 signaling enhances the expression of colony stimulating factor 1 receptor (CSF1R; also know as CD115), which is necessary for the development and maintenance of monocytes (35) and tissue macrophages (36). Therefore, CD18 may provide critical signals to maintain a functional CSF1-CSF1R axis in these cells during schistosomiasis. To test this hypothesis, we quantified the cell surface expression of CD115 in pMo and inflammatory Ly6C^high^ monocytes. As expected, the expression of CD115 was significantly reduced in the surface of pMo **(** **Figure 3C** **)** and Ly6C^high^ monocytes **(** **Figure 3D** **)** from *CD18^low^* mice at 7 wpi. To validate these findings and establish the impact of CD18 over CD115 expression, we treated naïve WT mice with anti-CD18 and analyzed CD115 expression in pMo and inflammatory Ly6C^high^ monocytes from the BM, according to the experimental design in **Figure 3E**. Compared to control mice, CD115 expression was reduced in the surface of pMo from anti-CD18 treated mice **(** **Figure 3F** **)**, while CD18 blockage did not change the CD115 expression in inflammatory Ly6C^high^ monocytes in the BM **(** **Figure 3G** **)**. Taken together, these results suggest that CD18 affects the IRF8-CD115 axis, which may modulate the proliferation of monocyte precursors and impact the generation of mature monocytes during chronic *S. mansoni* infection.

### CD18 regulates αM-CD11b subunit expression in hepatic Ly6C^high^ monocytes during schistosomiasis

Because CD18 partners with different α-subunits to compose distinct integrins such as α_L_β_2_ (CD11a/CD18 or LFA-1), α_M_β_2_ (CD11b/CD18 or Mac-1 or CR3), α_X_β_2_ (CD11c/CD18 or p150/95 or CR4) and α_D_β_2_ (CD11d/CD18) (1) and monocytes play a critical roles in regulating tissue damage (37), we investigated the gene expression of α-subunits in the liver from *S. mansoni-*infected WT mice at 7wpi. We found increased *Itgam* expression compared to *Itgal* **(** **Figure S2A** **)**. Additionally, we performed a flow cytometry analysis to evaluate the cell surface expression of α-subunits on CD45^+^ cells in the liver. The analysis revealed an increased frequency of CD11b^+^ cells compared to CD11a^+^ cells in the liver of *S. mansoni-*infected mice at 7 wpi **(** **Figures S2B, C** **)**. Accordingly, our previous work showed that, lower CD18 expression reduces the percentages of Ly6C^inter^ and patrolling Ly6C^low^ monocytes in the liver during chronic schistosomiasis (3). Therefore, we applied the flow cytometric gating hierarchy shown in **Figure S2B** to evaluate the α-subunits in hepatic inflammatory Ly6C^high^ and patrolling Ly6C^low^ monocytes. Compared to WT mice, CD11b and CD11c expression was reduced in inflammatory Ly6C^high^ monocytes in the livers of infected-*CD18^low^* mice, while α-subunits were not altered in patrolling Ly6C^low^ monocytes compared to both animals **(** **Figures S2D, E** **)**. Furthermore, we observed that the percentage of Ly6C^high^ CD11b^+^ monocytes was significantly diminished in the liver of *CD18^low^* mice **(** **Figure S2F** **)**. Of note, there were no significant differences in the percentage of CD11a^+^ or CD11c^+^ inflammatory Ly6C^high^ monocytes **(** **Figure S2F** **)** or any of patrolling Ly6C^low^ monocytes **(** **Figure S2G** **)**.

### Adoptive transference of inflammatory Ly6C^high^ monocytes ameliorate the liver damage in infected-*CD18^low^* mice during chronic schistosomiasis

**Figure 4:**
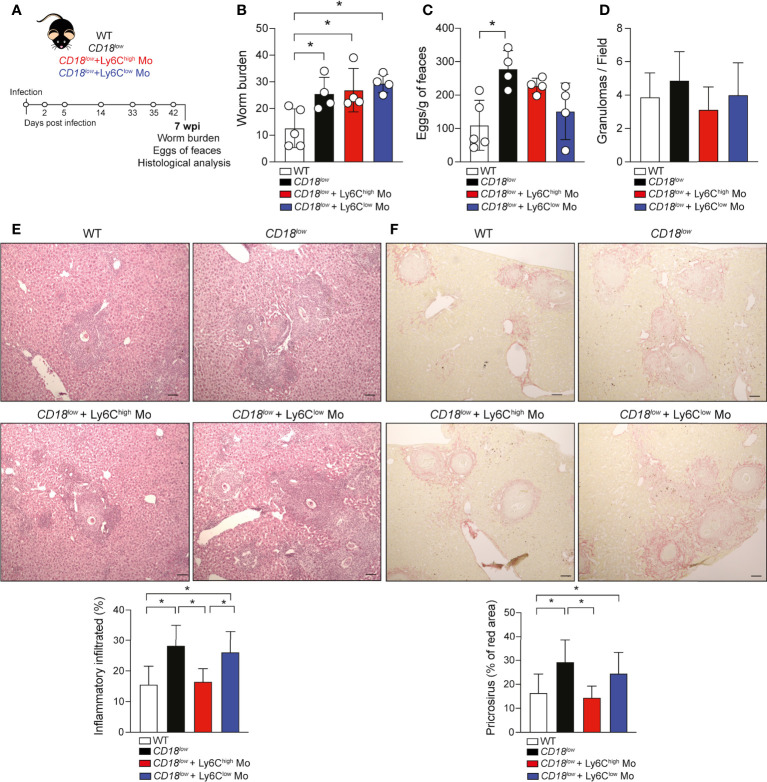
Adoptive transference of inflammatory Ly6C^high^ monocytes promotes the tissue repair in infected-*CD18^low^* mice during chronic schistosomiasis. WT and *CD18^low^* mice were subcutaneous infected with 80 cercariae of *S. mansoni*. **(A)** Schematic representation of the adoptive transference of inflammatory Ly6C^high^ CD11b^+^ and patrolling Ly6C^low^ CD11b^+^ monocytes previously isolated from uninfected *CX_3_CR1^gpf/wt^* reporter mice by FACS-sorting. **(B)** Scatter plots with bar show the parasite worm burden determined by perfusion of the hepatic portal system from WT and *CD18^low^* mice treated or not with inflammatory Ly6C^high^CD11b^+^ and patrolling Ly6C^low^ CD11b^+^monocytes at 7 wpi. **(C)** Scatter plots with bar show the eggs/g of feces from WT and *CD18^low^* mice treated or not with inflammatory Ly6C^high^ CD11b^+^ and patrolling Ly6C^low^ CD11b^+^at 7 wpi. **(D)** Bar plots show the numbers of granuloma by field from WT and *CD18^low^* mice receiving inflammatory Ly6C^high^ CD11b^+^ or patrolling Ly6C^low^ CD11b^+^ and controls at 7 wpi. **(E)** Photomicrographs of liver lesion stained with H&E coloration and bar plots show the percentage of the inflammatory infiltrated from WT and *CD18^low^* mice receiving inflammatory Ly6C^high^ CD11b^+^ or patrolling Ly6C^low^ CD11b^+^ and controls at 7 wpi. **(F)** Photomicrographs of liver lesion stained with picrosirius red staining and bar plots show the percentage of fibrosis (red area) from WT and *CD18^low^* mice receiving inflammatory Ly6C^high^CD11b^+^ or patrolling Ly6C^low^CD11b^+^ and controls at 7 wpi. All photomicrography was analyzed using a light microscope, scale bar: 50μM (n= 4 WT or *CD18^low^* control mice and n=4 *CD18^low^* adoptive transferred mice per group). Data are expressed as mean ± SD. Statistically significant differences were evaluated with ANOVA followed by Bonferroni’s multiple comparisons test (*p < 0.05).

To validate that CD18 is required by monocytes to protect from pathology, we isolated inflammatory Ly6C^high^CD11b^+^ or patrolling Ly6C^low^CD11b^+^ monocytes from a naïve CX_3_CR1-GFP reporter mice by FACS sorting, and transferred to infected *CD18^low^* mice, according to the experimental design in **Figure 4A**. Low CD18 expression results in increased worm burdens and release of eggs compared to WT mice **(** **Figures 4B, C** **)**. The adoptive transference of inflammatory Ly6C^high^CD11b^+^ or Ly6C^low^CD11b^+^ monocytes to infected-*CD18^low^* mice did not impact these parameters at 7 wpi **(** **Figures 4B, C** **)**. Inflammatory Ly6C^high^ monocytes reduce Ly6C expression and accumulate in the hepatic granulomas around the *S. mansoni* eggs (6, 15, 16), but low CD18 expression does not affect granuloma areas in responses to *S. mansoni* eggs (3). Thus, we performed histological analyses of livers from both groups of mice infected at 7 weeks by staining with hematoxylin & eosin (H&E) and picrosirius red to evaluate the inflammatory infiltrated and fibrosis, respectively. The numbers of granulomas were similar between WT and *CD18^low^* mice receiving Ly6C^high^CD11b^+^ or Ly6C^low^CD11b^+^ monocytes and controls **(** **Figure 4D** **).** Compared to WT, *CD18^low^* mice displayed increased inflammatory infiltrate in the liver **(** **Figure 4E** **)** and the adoptive transference of WT inflammatory Ly6C^high^ CD11b^+^ monocytes to infected-*CD18^low^* reduced the inflammatory infiltrate in the liver, which became comparable to WT mice **(** **Figure 4E** **)**. There were no significant differences in mice receiving WT patrolling Ly6C^low^CD11b^+^ monocytes **(** **Figure 4E** **)**. Consistently, we observed increased collagen deposition in livers of *CD18^low^* compared to WT mice **(** **Figure 4F** **)**, while *CD18^low^* mice that received WT inflammatory Ly6C^high^ monocytes exhibited diminished hepatic fibrosis **(** **Figure 4F** **)**. Adoptive transference of WT patrolling Ly6C^low^ CD11b^+^ monocytes had no effects to control the fibrosis in liver (**Figure 4F**). Overall, these data show that CD18 is critical for the generation of inflammatory Ly6C^high^ monocytes, which are required to control the inflammation and fibrosis in liver during chronic schistosomiasis.

### The alternative activation of macrophages requires CD18 during chronic schistosomiasis

**Figure 5:**
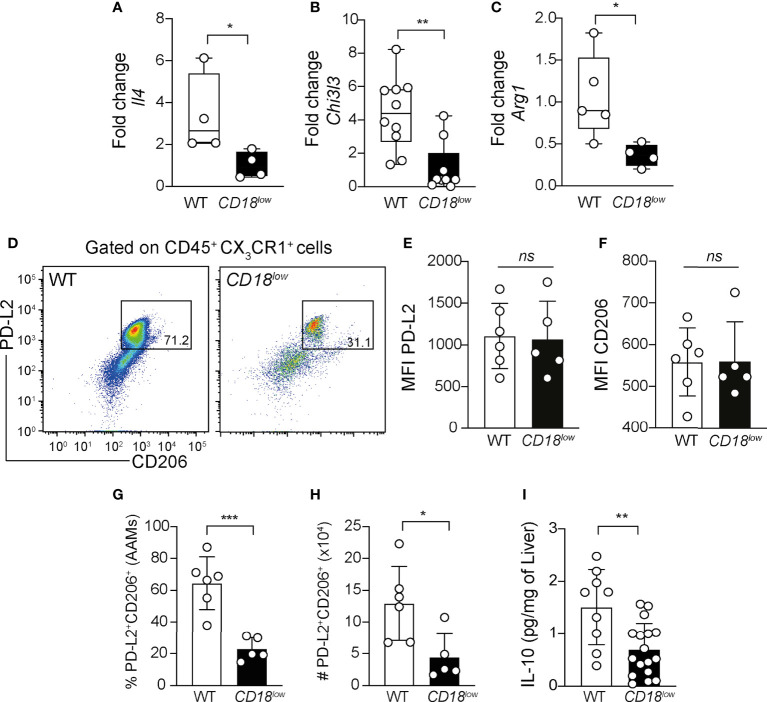
Low CD18 expression impacts alternative activation of macrophages in livers of *S. mansoni*-infected mice. WT and *CD18^low^* mice were subcutaneous infected with 80 cercariae of *S. mansoni* and the livers were collected at 7 wpi. Graphs display the qRT-PCR analysis of *Il4* **(A)** *Chil3l3* **(B)** and *Arg1* **(C)** expression in the liver. Data were analyzed from one representative experiment **(A, C)** or pool **(B)** out of two independent experiments. Statistically significant differences were evaluated with Mann-Whitney test (*p< 0.05, **p<0,01 compared to WT in each-time point). **(D)** Representative dot plots display the flow cytometric data of CD45^+^CX_3_CR1^+^PL-D2^+^CD206^+^ alternatively activated macrophages (AAMs). **(E, F)** Scatter plot with bar show the median fluorescent intensity (MFI) of PD-L2. **(F)** and CD206 **(F)** in AAMs. **(G, H)** Scatter plot with bar show the percentage **(G)** and absolute numbers **(H)** of these cells in the liver. Median with interquartile range is shown for one representative experiment (n= 6 WT and n=5 *CD18^low^* infected mice at 7 weeks) out of three independent experiments. Data were analyzed with Mann-Whitney test (* p < 0.05, *** p < 0.001, ns p > 0.05, compared to WT mice in each time-point). **(I)** Scatter plot with bar show the levels of IL-10 by ELISA in the liver. Data are from a pool of three independent experiments and were analyzed with Mann-Whitney test (**p < 0.01 compared to WT mice). The symbol (#) indicates the absolute numbers of AAMs.

Ly6C^high^ monocytes give rise to AAM in liver granulomas, which are key players of tissue damage repair and granuloma formation during schistosomiasis (6, 15). To understand whether CD18 is required for the polarization and function of AAM, we evaluated the expression of genes characterizing the alternative activation of macrophages in the liver from WT and *CD18^low^* mice at 7 wpi. Compared to WT mice, the expression of *Il4* **(** **Figure 5A** **)**, *Chi3l3* **(** **Figure 5B** **)** and *Arg1* **(** **Figure 5C** **)** was reduced in *CD18^low^* animals. Next, we evaluated the accumulation of AAM in the liver using the flow cytometric gating hierarchy shown in **Figure S3**. There were no significant differences in PD-L2 and CD206 expression on AAMs between WT and *CD18^low^* mice **(** **Figures 5D–F** **).** However, both percentage and absolute numbers of PD-L2^+^CD206^+^ AAMs decreased in the liver of *CD18^low^* mice compared to WT animals at 7 weeks **(** **Figures 5D, G, H** **)**. IL-4Ra signaling in AAM induces IL-10 production which is necessary for the maintenance of granulomas around the eggs (38). We observed that *S. mansoni-*infected *CD18^low^* mice displayed reduced abundance of IL-10 in the liver **(** **Figure 5I** **)**, thus the reduced AAM population may reflect into lower IL-10 levels. Taken together, these data suggest that CD18 affects the phenotype and function of AAM during chronic schistosomiasis.

## Discussion

Our study showed that low levels of CD18 affect pMo progenitor cells, which accumulate in the BM, while reducing mature monocytes, immune cells that play critical roles during schistosomiasis (39). The data also suggest that CD18, and potentially the integrin Mac-1, regulates the gene expression of *Irf8*, which in turn is required for CD115 expression. Without optimal CD115 signaling, pMo may fail to mature into inflammatory Ly6Chigh monocytes, which are required to protect from immunopathology and death (3). Previous studies suggest that CD18 influences the formation of hematopoietic niches (11, 12) by triggering the proliferation and differentiation of HSPCs, which lead to myeloid (3, 9, 40, 41) and lymphoid cells hematopoiesis (42). It is important to note that proteomic analysis of monocytes and their progenitors showed that ITGB2 is involved in the discrimination between the developmental stages (14). Additionally, naïve CD18^-/-^ mice display accumulation of granulocyte-macrophages progenitor (GMP) cells in the BM, which is associated with a higher GATA2 expression and activation of IgE – FcϵRI axis leading the GMP expansion and granulocyte and monocyte production (9). Curiously, arteriosclerotic progression has been associated with HSPC expansion in the BM and their recruitment to injured arteries *via* CD18 expression (40). In line with this metabolic disorder, *S. mansoni-*infected male Apolipoprotein E deficient (*ApoE^-/-^*) mice on high-fat diet (HFD) modulates oxygen consumption on bone marrow myeloid progenitors, culminating in Ki-67 expression and expansion of GMP (43). Here, we found that low levels of CD18 reduced Ki-67 expression in monocyte precursors, which suggests lower proliferation and maturation of these cells during *S. mansoni* infection, but further studies are necessary to confirm the precise biochemical pathways by which CD18 controls monocytopoiesis during schistosomiasis.

Impaired development of monocytes has been correlated with defective mTOR signaling, which results in the activation of STAT5 and downregulation of IRF8-dependent CD115-expression in myeloid progenitor cells in the BM (30). IRF8-deficient mice display reduced frequency and numbers of mature monocytes in BM and blood, while the IRF8-transduced cells increase *Itgam, Csfr1* and *Cd14* expression (32). Furthermore, IRF8 signaling modulates the effector functions of macrophages and dendritic cells by increasing the cell surface expression of other integrin families such as β8 and β7 (44). We observed a significant reduction of IRF8 gene expression in pMo of *S. mansoni*-infected *CD18^low^* mice, which also displayed reduced CD115 expression in their surface. Of interest, CD115 expression was also reduced in inflammatory Ly6C^high^ monocytes. By blocking CD18 *in vivo* in naïve WT animals, we confirmed that CD18 is required for CD115 expression in pMo, but not inflammatory Ly6C^high^ monocytes, emphasizing their importance for monocyte development and maturation even in absence of infection. However, limitations of our study include whether Mac-1 mediates the intracellular cascades regulating IRF8 at the transcriptional level and whether reduced levels of IRF8 indeed cause lower CD115 expression and signaling in pMo during schistosomiais.

Once they infiltrate into inflammatory tissue, monocytes and macrophages mediate effector responses to *S. mansoni* eggs and contribute to granuloma formation together with other immune and non-immune cells (39). In the tissue, Mac-1 integrin (CD11b/CD18) is required for effective macrophage responses and tissue remodeling (45). The sensibilization with soluble eggs antigen (SEA) of *S. mansoni* induces high expression of Mac-1 that correlates with reduction of liver fibrosis (46). Overall, our data demonstrate that low CD18 expression affects the maturation of inflammatory Ly6C^high^CD11b^+^ monocytes, while adoptive transference of these cells, but not patrolling monocytes, ameliorated the inflammatory infiltrate and fibrosis in liver from *S. mansoni-* infected *CD18^low^* mice. Ideally, adoptive transfer of pMo progenitors from WT mice would better reveal their contribution in this context, while, adoptive transfer of inflammatory monocytes and/or pMo progenitors from *CD18^low^* mice donors shell confirm the requirement of CD18 by inflammatory monocytes for the polarization of AAM. In support of our findings, cellular therapy of monocytes (CD14^+^ CD11b^+^) contributes to tissue remodeling, reduces the production of TGF-β and upregulates *Fizz1* (M2 marker) expression in the liver during experimental schistosomiasis (37).

During *S. mansoni* infection, IL-4Rα signaling is dispensable to blood monocyte influx and their conversion to MDM (16). However, IL-4 and IL-13 trigger the extracellular matrix remodeling and promote the alternative activation of macrophages (6, 15, 39, 47), while the alternative activation is inhibited by the direct interaction of Mac-1 integrin with IL-13Rα1 on the cellular surface (26). In contrast our data suggest that Mac-1 is needed for the alternative activation of macrophages during *S. mansoni* infection. We observed a decreased expression of genes related to AAM, reduced frequency and numbers of PD-L2^+^CD206^+^ AAMs, and decreased levels of IL-10 in the liver from *S. mansoni*-infected *CD18^low^* mice. AAM limit liver and intestinal fibrosis caused by deposition of eggs, becoming essential to host protection against pathology caused by *S. mansoni* infection (48). Interestingly, IL-10^-/-^ mice infected with *S. mansoni* display higher pathology and less well-defined granulomas (49) while patients with severe schistosomiasis produce lower levels of IL-10 than those without fibrosis (50).

The data suggest a model in which CD18, and possibly Mac-1 integrin, induce the proliferation of monocyte progenitors as well as the IRF8 - CD115 axis. This could be required for the maturation of monocytes in the bone marrow. Furthermore, the data suggest that CD18 is needed for the phenotype and function of AAM, which would impact not only schistosomiasis, but also other helminthic, infectious and inflammatory diseases.

## Data availability statement

The original contributions presented in the study are included in the article/**Supplementary Material**. Further inquiries can be directed to the corresponding author.

## Ethics statement

The animal study was reviewed and approved by The Comissão de Ética no Uso de Animais da Faculdade de Ciências Farmacêuticas de Ribeirão Preto (Protocol Number 14.1.607.53.9 and 19.1.46.60.4) and carried out in accordance to the ethical principles for animal research adopted by the Sociedade Brasileira de Ciência em Animais de Laboratório.

## Author contributions

CS and LF conceived the study. CS, JE-O, MP, CF, VR and LG performed experiments. CS conduced data analysis and wrote the original draft. CS and LG reviewed and edited the paper. CS, LG and LF inputted intellectual concepts. VR maintained parasites and provided infection model. LF provide resources and coordinated the study. All authors read and approved the final manuscript.

## Funding

This work was supported by São Paulo Research Foundation (Fundação de Amparo à Pesquisa do Estado de São Paulo (FAPESP), Grant n. #2014/07125-6 to LHF; and scholarship #2018/22667-0 to COSS); the Conselho Nacional de Desenvolvimento Científico e Tecnológico (CNPq) Grant n. #302514/2015-5, #408093/2018-8 and #303259/2020-5), and Coordenação de Aperfeiçoamento de Pessoal de Nível Superior (CAPES) – Final Code 001. LGG and LHF are research fellows from the CNPq.

## Acknowledgments

We are thankful to Fabiana Rosseto de Moraes and Denize Ferraz for help us with the acquisition of flow cytometry data and FACS-cell sorting, respectively. Elaine Medeiros Floriano for the help with histology staining. Izaira Tincani Brandão and Viviani Nardini Takahashi for the help with technical assistance. Ronaldo Araujo, Fabio Junior Marsola and Reinaldo Fernando Bastista for animal maintenance and all and Dr. Faccioli’s laboratory members for their scientific discussion and insightful comments. We also thank Lizandra Guidi Magalhões, Ph.D. for helping with *S. mansoni* life cycle during COVID-19 pandemic.

## Conflict of interest

The authors declare that the research was conducted in the absence of any commercial or financial relationships that could be construed as a potential conflict of interest.

## Publisher’s note

All claims expressed in this article are solely those of the authors and do not necessarily represent those of their affiliated organizations, or those of the publisher, the editors and the reviewers. Any product that may be evaluated in this article, or claim that may be made by its manufacturer, is not guaranteed or endorsed by the publisher.
