# Supplementary material for: CD18 controls the development and activation of monocyte-to-macrophage axis during chronic schistosomiasis
Source: Front Immunol. 2022 Oct 3;13:929552. doi: 10.3389/fimmu.2022.929552 (PMC9574367; doi:10.3389/fimmu.2022.929552)
Supplement: Supplementary file 1 [file DataSheet_1.docx]

Supplementary Material

CD18 controls the development and activation of monocyte-to-macrophage axis during chronic schistosomiasis

Camila O. S. Souza^1,2^, Jefferson Elias-Oliveira^1,2^, Marcella R. Pastore^1,3^, Caroline Fontanari^1^, Vanessa F. Rodrigues^4^ ,Vanderlei Rodriguez^4^, Luiz G. Gardinassi^5^ and Lúcia H. Faccioli^1^

*** Correspondence:**Lúcia Helena Faccioli
[faccioli@fcfrp.usp.br](mailto:faccioli@fcfrp.usp.br)


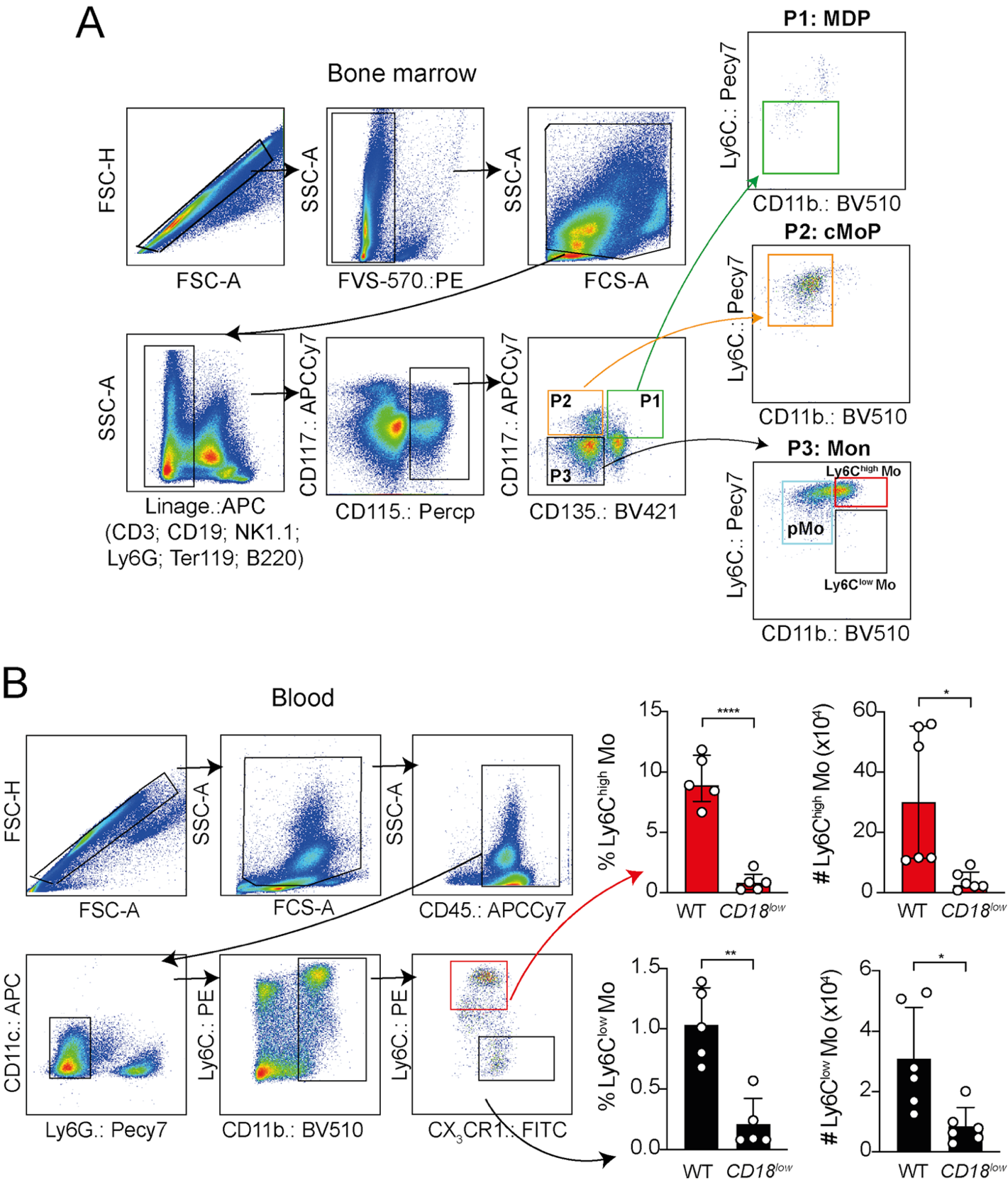


**Supplementary Figure 1.** **Low CD18 expression affects the monocytopoiesis during chronic schistosomiasis. (A)** Dot plots show representative flow cytometric gating hierarchy for analysis monocytes progenitor cells and monocytes subsets in Lin- (CD3, CD19, NK1.1, Ly6G, Ter199, B220) from the bone marrow. Plots are include in Figure 1. **(B)** Dot plots show representative flow cytometric gating hierarchy for analysis inflammatory Ly6C^high^ and patrolling Ly6C^low^ monocytes and scatter plot with bar show the percentage and absolute numbers of these cells in the peripheral blood. Data are from a pool of two independent experiments and were analyzed with Mann-Whitney test (*p< 0,05, **p < 0.01, ****p<0,0001 compared to WT in each-time point).


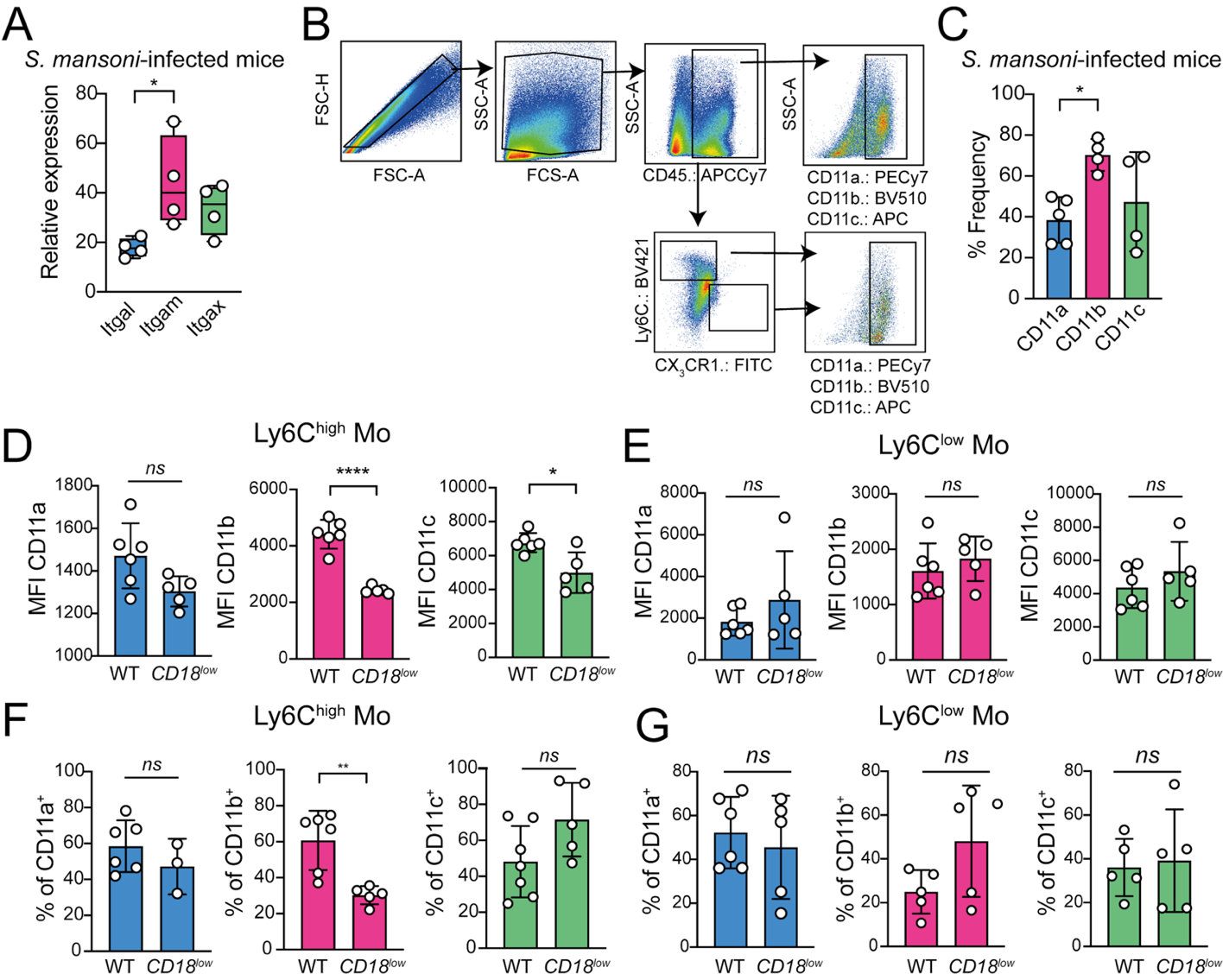


**Supplementary Figure 2. Low CD18 expression affects the α-subunit CD11b in livers of *S. mansoni-*infected mice.** Livers of uninfected and *S. mansoni-*infected C57BL/6 (WT) and *CD18^low^* mice were collated at 7 wpi. **(A)** Graphs display the expression of *Itgal*, *Itgam* and *Itgax* measured by qRT-PCR from *S. mansoni-*infected mice at 7 weeks. Data are from one independent experiment (n=4 WT mice) and were analyzed with Kruskal-Wallis followed by Dunn’s multi-comparison test (*p< 0,05 compared between α-subunit). **(B)** Contour plots show representative flow cytometric gating hierarchy for analysis of FSC-H/FSC-A, SSC-A/FSC-A followed CD45^+^, CD11a^+^, CD11b^+^, CD11c^+^ (upper panel) or monocytes subsets and subsequent CD11a^+^, CD11b^+^ CD11c^+^ for each monocyte subset (bottom panel). **(C)** Scatter plot with bar show the percentage of CD11a^+^, CD11b^+^ CD11c^+^ in total CD45^+^ leukocytes from *S. mansoni*-infected mice at 7 weeks. **(D – E)** Scatter plot with bar show the median fluorescent intensity (MFI) of CD11a, CD11b, CD11c in inflammatory Ly6C^high^ CX_3_CR1^low^ **(D)** and patrolling Ly6C^low^ CX_3_CR1^high^ **(E)** monocytes. **(F – G)** Scatter plot with bar show the percentage of inflammatory Ly6C^high^ CX_3_CR1^low^ **(F)** and patrolling Ly6C^low^ CX_3_CR1^high^ **(G)** monocytes that expressed CD11a^+^, CD11b^+^ and CD11c^+^ in liver leukocytes from WT and *CD18^low^ mice* at 7 wpi with *S. mansoni*. Median with interquartile range are shown for one representative experiment (n= 5-6 WT and n=3-5 *CD18^low^* infected mice at 7 weeks) out of three independent experiments. Data were analyzed with Mann-Whitney test (*p < 0.05 compared to WT mice in each time-point). **(F)** Dot plots show representative flow cytometric gating hierarchy for analysis of FSC-H/FSC-A, SSC-A/FSC-A followed CD45^+^, CX_3_CR1^+^, PD-L2^+^ CD206^+^ alternatively activated macrophages (AAM) in the liver.


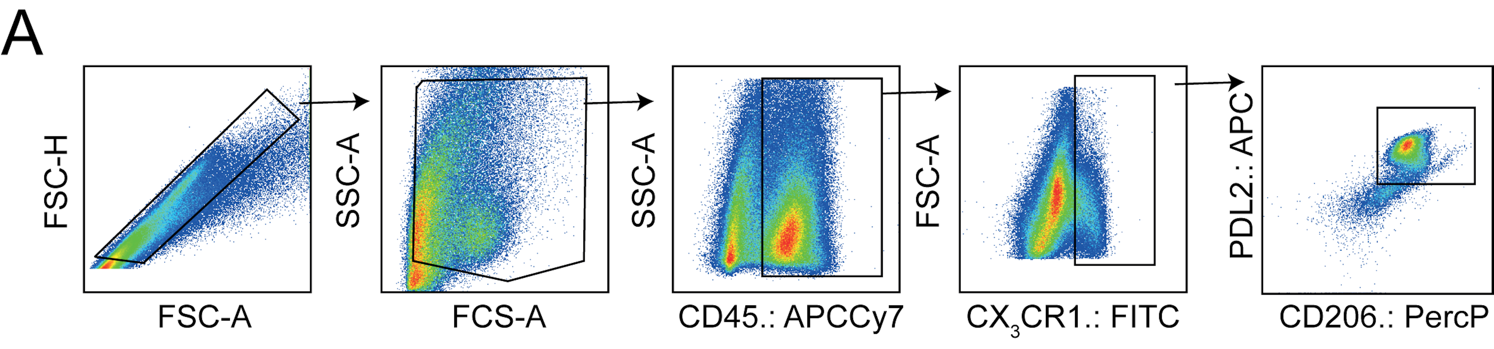


**Supplementary Figure 3. Flow cytometry gating strategy for alternatively activated macrophages. (A)** Contour plots show representative flow cytometric gating hierarchy for analysis of FSC-H/FSC-A, SSC-A/FSC-A followed CD45^+^, CX3CR1, PD-L2, CD206 alternatively activated macrophages.

**Supplementary Table 1. Murine primer sequences used in the study**

| **Gene** | **Forward (5'-3')** | **Reverse** |
| --- | --- | --- |
| *Gapdh* | CGA AGG TGG AAG AGT GGG AG | TGA AGC AGG CAT CTG AGG G |
| *Irf8* | CAG ATC CTC CCT GAC TGG TG | GCT TGC CCC CGT AGT AGA AG |
| *Klf4* | GAG GCT GTG GCA AAA CCT AT | CGG TAG TGC CTG GTC AGT TC |
| *Nr4a1* | AGC TTG GGT GTT GAT GTT CC | AAT GCG ATT CTG CAG CTC TT |
| *Itgal* | TCC GGA AAG TGG AGA TGC TT | GAA GTC TTC CCA GGA GCT GT |
| *Itgam* | TCC GGT AGC ATC AAC AAC | GGT GAA GTG AAT CCG GAA CT |
| *Itgax* | CTG GAT AGC CTT TCT TCT GCT G | GCA CAC TGT GTC CGA ACT CA |
| *Il4* | AAG AGC ATC ATG CAA ATG GA | TTA AAG CAT GGT GGC TCA GTA |
| *Chi3l3* | AGA AGG GAG TTT CAA ACC TGG T | GTC TTG CTC ATG TGT GTA AGT GA |
| *Arg1* | CTC CAA GCC AAA GTC CTT AGA G | AGG AGC TAT CAT TAG GGA CAT C |
